# MSMTRIU-Net: Deep Learning-Based Method for Identifying Rice Cultivation Areas Using Multi-Source and Multi-Temporal Remote Sensing Images

**DOI:** 10.3390/s24216915

**Published:** 2024-10-28

**Authors:** Manlin Wang, Xiaoshuang Ma, Taotao Zheng, Ziqi Su

**Affiliations:** 1 School of Resources and Environmental Engineering, Anhui University, Hefei 230601, China; x02014128@stu.ahu.edu.cn (M.W.); x02014066@stu.ahu.edu.cn (T.Z.); x02014174@stu.ahu.edu.cn (Z.S.); 2 Information Materials and Intelligent Sensing Laboratory of Anhui Province, Anhui University, Hefei 230601, China; 3 Engineering Center for Geographic Information of Anhui Province, Anhui University, Hefei 230601, China

**Keywords:** multi-source remote sensing, agriculture remote sensing, deep learning, rice monitoring, feature fusion

## Abstract

Identifying rice cultivation areas in a timely and accurate manner holds great significance in comprehending the overall distribution pattern of rice and formulating agricultural policies. The remote sensing observation technique provides a convenient means to monitor the distribution of rice cultivation areas on a large scale. Single-source or single-temporal remote sensing images are often used in many studies, which makes the information of rice in different types of images and different growth stages hard to be utilized, leading to unsatisfactory identification results. This paper presents a rice cultivation area identification method based on a deep learning model using multi-source and multi-temporal remote sensing images. Specifically, a U-Net based model is employed to identify the rice planting areas using both the Landsat-8 optical dataset and Sentinel-1 Polarimetric Synthetic Aperture Radar (PolSAR) dataset; to take full into account of the spectral reflectance traits and polarimetric scattering traits of rice in different periods, multiple image features from multi-temporal Landsat-8 and Sentinel-1 images are fed into the network to train the model. The experimental results on China’s Sanjiang Plain demonstrate the high classification precisions of the proposed Multi-Source and Multi-Temporal Rice Identification U-Net (MSMTRIU-NET) and that inputting more information from multi-source and multi-temporal images into the network can indeed improve the classification performance; further, the classification map exhibits greater continuity, and the demarcations between rice cultivation regions and surrounding environments reflect reality more accurately.

## 1. Introduction

In the new era, global population growth and climate change impose greater pressures on food supply [1,2]. Rice, a pivotal staple crop, serves a vital role for food security for more than half of the world’s population [3,4]. The timely and accurate monitoring of rice cultivation areas holds great significance in comprehending the overall distribution pattern of rice and formulating agricultural policies. The traditional statistical methods resort to sampling surveys and hierarchical reporting, both of which involve large costs in data acquisition [5]. Remote sensing technology, with its advantages in terms of the frequent acquisition of spectral information for land objects, offers significant benefits in terms of large coverage and cost-effectiveness for Earth observations [6,7]. Presently, it has become a crucial technological tool for extracting spatial distribution information of rice [8].

Currently, most research on rice monitoring relies on optical remote sensing observations. One typical class of methods is to use various vegetation indices for rice map-ping by considering the phenological characteristics of rice growth periods. Gumma et al. [9] utilized MODIS data to generate seasonal maximum NDVI time series for extracting information on rice planting areas across three seasons in Bangladesh. In the literature [10], Dan et al. proposed the rice zoning adaptability criteria and dynamic harvest index (RZAC-DHI) by employing an enhanced regional parametric syntheses approach. Another typical class of methods for rice monitoring are machine learning-based methods. For example, Triscowati et al. [11] developed a classification model of the rice-plant phase by using Landsat 8 satellite imagery. Taking the Nganjuk area in East Java, Indonesia as a case study, Saadi et al. [12] investigated the performance in detecting rice production areas using Sentinel-2 and Landsat-8 images by incorporating a range of machine learning classifiers and multiple spectral indices. In recent years, due to their ability to automatically mine features for specific classification tasks without the need for a manual feature extraction process, deep learning methods have attracted wide attention in many fields. A typical application of a deep learning method is medical image segmentation. For example, in [13], a multi-task, multi-scale learning framework for predicting the patient’s survival and response was established. Deep learning methods have also been applied for rice identification on remote sensing images. Li et al. [14] used multi-temporal Sentinel-2 images to accurately delineate crop distributions by employing advanced 3D-CNN and ConvLSTM2D models, which utilize multiple vegetation indices. In literature [15], Xia et al. constructed a large-scale Landsat 8 dataset and full resolution network for rice classification in northern China.

Although optical remote sensing observation can capture rich spectral reflection information of crops, cloud cover often hinders the acquisition of high-quality optical images. Thanks to the capability of acquiring images in all-weather conditions, Polarimetric Synthetic Aperture Radar (PolSAR) technology has been increasingly utilized in monitoring rice in recent years. Corcione et al. [16] used X-band dual-polarimetric Cosmo-SkyMed data to monitor rice growth conditions in the Mekong Delta region of Vietnam and found that the polarization ratio between HH and VV can effectively distinguish the four growth stages of rice. Son et al. [17] employed random forest and support vector machine methods for mapping rice-cropping systems in South Vietnam by using the smoothed temporal sequences of VH backscattering data from Sentinel-1A images. A large-scale paddy rice mapping strategy was proposed, utilizing an enhanced U-Net model to generate annual paddy rice maps of Thailand using time-series SAR images [18].

Although the studies mentioned above indicate that the use of optical or PolSAR remote sensing images in rice monitoring is promising, there are still some improvements that can be made, which are as follows: (1) optical images can provide rich spectral reflection information of crops, while PolSAR images can provide rich polarimetric scattering information of crops. Thus, in theory, by combining multi-source images, more radiometric information of rice can be mined to improve the identification accuracy. In some certain periods, rice may have similar spectral traits as other plants. Therefore, considering the spectral information of rice at different growth stages can help distinguish it from other plants. However, to our knowledge, most studies have not utilized the images from different type of sensors and different imaging times, making the identification results not robust in some cases. (2) Although some research has shown the tremendous potential of utilizing PolSAR images for monitoring paddy rice, most of them focused on using only amplitude or intensity (the square of amplitude) data and ignoring other polarimetric information (for example, the polarimetric phase), leading to unsatisfactory performances in distinguishing crops that have similar backscattering amplitudes.

Starting from the above viewpoints, this paper presents a U-Net deep learning-based model to identify rice cultivation areas by integrating the spectral reflection information of rice from the Landsat-8 optical image and the polarimetric scattering information of rice from Sentinel-1 dual PolSAR; to take full account of the information in different periods, multiple image features from multi-temporal images are fed into the network to train the model.

This work is structured as follows: Section 2 describes the study area, dataset, and data-processing methods. Section 3 introduces the Multi-Source and Multi-Temporal Rice Identification U-Net (MSMTRIU-NET). Section 4 presents the experimental results, and Section 5 concludes the paper.

## 2. Study Area and Datasets

### 2.1. Study Area

**Figure 1:**
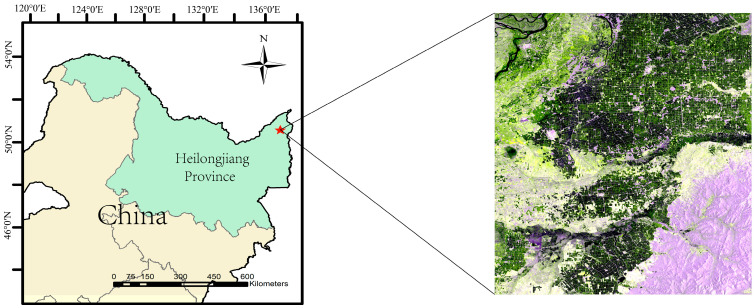
The map of the study area.

The study area is located in the northeastern region within China’s Sanjiang Plain. Sanjiang Plain is located approximately between 45° N~39° N and 130° E~135° E, primarily shaped by the sedimentation of the Heilongjiang, Wusuli, and Songhua Rivers, as shown in Figure 1. Additionally, Sanjiang Plain is renowned for its flat terrain and fertile soil, with a mild and humid continental monsoon climate that creates ideal conditions for agricultural cultivation. The study area is dedicated to the cultivation of early-maturing, single-crop rice, typically sown in early-April to mid-April each year and harvested from September to October, with the growth cycle lasting approximately 200 to 240 days. Currently, it has achieved a highly mechanized agricultural production system with a large production amount of rice. This accomplishment ensures a highly synchronized process throughout the entire cycle of rice cultivation and harvesting in the region. This high degree of synchronization significantly enhances the feasibility of utilizing multi-source and multi-temporal remote sensing imagery for the recognition of large-scale rice cultivation areas.

### 2.2. Remote Sensing Dataets and Preprocessing

**Figure 2:**
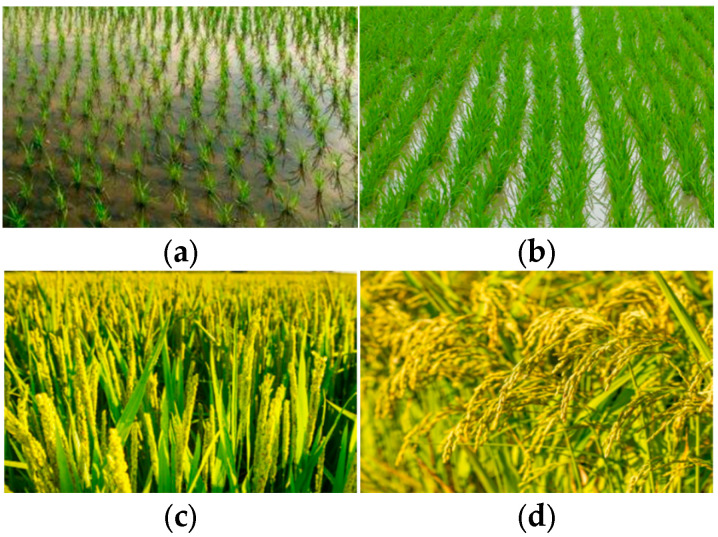
Photographs for the different phenological stages of rice: (**a**) Seedling stage; (**b**) Tillering stage; (**c**) Heading stage; (**d**) Maturity stage.

Optical and PolSAR images are employed across various rice growth seasons to identify rice cultivation areas in the study areas in 2020—namely, the seedling stage, tillering stage, heading stage, and maturity stage, according to the morphological alterations and growth characteristics exhibited by rice during its lifecycle as shown in Figure 2. For certain periods, the spectral reflectance and polarimetric scattering characteristics of rice may exhibit similarities with some other vegetation types. Therefore, images from different phenological stages are necessary to distinguish rice from other vegetation types.

Successfully launched on 11 February 2013, Landsat-8 serves as an optical satellite for Earth observation. Equipped with the Operational Land Imager (OLI) and the Thermal Infrared Sensor (TIRS), this satellite can conduct comprehensive observations of the Earth [19,20]. As displayed in Table 1, the spectral information from seven bands of OLI is utilized for rice identification. Landsat-8 images can be freely accessed via the USGS Earth Explorer platform (https://earthexplorer.usgs.gov, accessed on 8 May 2022). To minimize the influence brought by cloud coverage, we only select Landsat-8 images with little cloud coverage. The preprocessing steps (including radiometric calibration, atmospheric correction, and resampling) are deployed.

The Sentinel-1 system is a dual-satellite C-band SAR system, with the Sentinel-1A satellite launched on 3 April 2014 and the Sentinel-1B satellite launched on 25 April 2016. The spatial resolution of a Sentinel-1 image is 10 m × 10 m. The Sentinel-1 system can acquire an image with two polarization modes: VV (Vertical-Vertical) and VH (Vertical-Horizontal). In this study, the Single Look Complex (SLC) Sentinel-1 remote sensing data with VV and VH polarizations are used [21]. The data can be freely downloaded from the European Space Agency Data Distribution website (https://search.asf.alaska.edu/#/, accessed on 15 May 2022). Table 2 provides an overview of the basic product information of Sentinel-1. For Sentinel-1 SAR images, geographical correction and speckle filtering are two key preprocessing steps, which can be implemented by the SANP software (Version 5.0.0).

**Figure 3:**
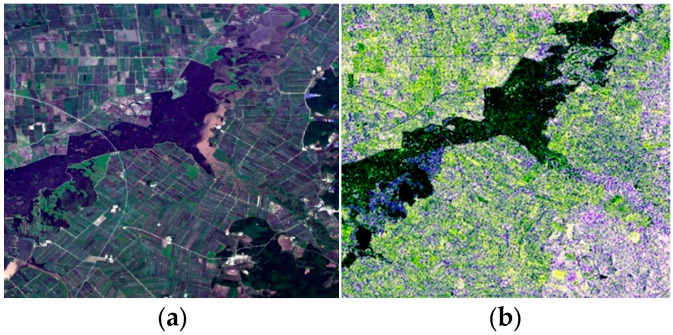
Multi-source images of the study area: (**a**) Lansat-8 image; (**b**) Sentinel-1 image.

In principle, to fully mine the phenological characteristics of rice, a multi-temporal remote sensing dataset needs to be constructed which includes, at least, one optical image and one PolSAR image in each growing stage. However, due to the influence of clouds, we can only acquire high-quality optical images of the study area in the heading stage and maturity stage in 2020. Table 3 lists the multi-temporal and multi-source remote sensing dataset used in this study. Figure 3 shows the Landsat-8 image and the Sentinel-1 image acquired on 3 July 2020.

### 2.3. Polarimetric Decomposition of Dual-Polarization SAR Data

As a representation of the information contained within each pixel of polarimetric radar imagery, a polarimetric covariance matrix is often used, which is formulated as

(1) $$C_{2\times 2}=\left[\begin{array}{cc} C_{11} & C_{12}\\ C_{21} & C_{22} \end{array}\right]=\left[\begin{array}{cc} \langle 2{\left.{\left|S\right.}_{HV}\right|}^{2}\rangle & \left\langle \sqrt{2}\right.\left.S_{HV}S_{VV}^{*}\right\rangle \\ \left\langle \sqrt{2}\right.\left.S_{VV}S_{HV}^{*}\right\rangle & \langle {\left.{\left|S\right.}_{VV}\right|}^{2}\rangle \end{array}\right]$$

with

(2) $$S_{HV}=\left|S_{HV}\right|e^{j\varphi_{HV}}$$

where $\left.{\left|S\right.}_{HV}\right|$ and $\varphi_{HV}$ represent the amplitude and the phase of the polarization channel with the transmitted horizontal (H) polarized wave and the received vertical (V) polarized wave, respectively; j is the imaginary unit [22].

Although some studies have shown the feasibility of using PolSAR images for monitoring rice, most of them focused on using only amplitude and ignoring other polarimetric information, leading to unsatisfactory performance in distinguishing crops which have similar backscattering amplitudes. In this study, all elements of the above matrix are used in the model, that is to say, both amplitude information and phase information are used.

Besides amplitude and phase, polarimetric decomposition parameters are also employed in the model. Polarimetric target decomposition theory can well reflect the polarimetric scattering mechanisms of targets, which play a crucial role in enhancing the precision of target detection, classification, and the retrieval of physical properties in many applications. This study employed the polarimetric entropy/polarimetric anisotropy/alpha angle (H/A/Alpha) polarimetric decomposition method [23,24,25].

The polarization entropy H can be expressed as follows:

(3) $$H=-\sum_{i=1}^{3}p_{i}{log}_{3}\left(p_{i}\right)$$

with

(4) $$p_{Z}=\frac{\lambda_{1}}{\lambda_{1}+\lambda_{2}},\;\lambda_{1}>\lambda_{2}$$

The anisotropy parameter A can be expressed as follows:

(5) $$A=\frac{\lambda_{1}-\lambda_{2}}{\lambda_{1}+\lambda_{2}}$$

The average scattering angle *α* can be expressed as

(6) $$\alpha =\sum_{i=1}^{3}p_{i}\alpha_{i}$$

The range of values for *H* is [0, 1], representing the degree of randomness in scattering, with lower H values indicating lower scattering randomness. H exhibits a higher sensitivity to the plant stem height. The range of values for *A* is [0, 1], reflecting the relationship between the two scattering mechanisms corresponding to $\lambda_{1}$ and $\lambda_{2}$. The range of values for α is [0°, 90°], and it is associated with the scattering mechanisms of the target. Both H and α are sensitive to the stem height of crops [26,27].

**Figure 4:**
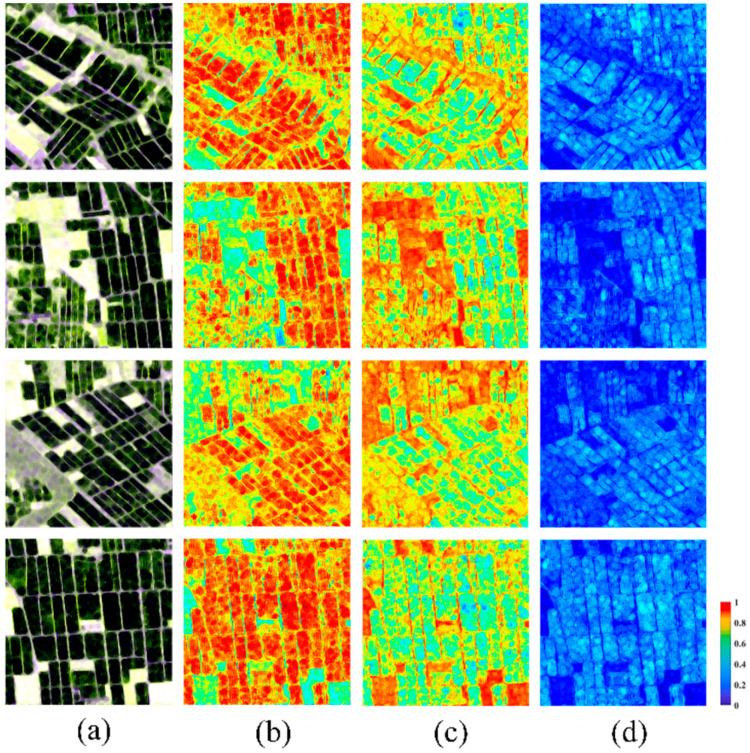
Polarimetric decomposition images of the rice cultivation areas: (**a**) Original Sentinel-1 images; (**b**) Corresponding polarimetric entropy images; (**c**) Corresponding polarimetric anisotropy images; (**d**) Corresponding polarimetric alpha angle images.

Some polarimetric decomposition images of the paddy rice cultivation areas in the study area are shown in Figure 4. In the corresponding polarimetric entropy images, rice cultivation areas show higher values. This is because signals from background targets (bare lands) primarily result from odd scattering, whereas the scattering characteristics of rice cultivation areas are more intricate. In the corresponding polarimetric anisotropy images, background targets exhibit higher values. This is due to the scattering contribution of the second dominant scattering mechanism in the polarimetric matrix being much lower than that of the most dominant scattering mechanism. In the corresponding polarimetric alpha angle images, rice cultivation areas exhibit higher values. This is because the presence of plant canopy structures results in more returns of double-bounce scattering signals, while bare lands are with fewer double-bounce scattering signals. The above conclusion indicates that incorporating polarimetric decomposition parameters into the input part of the proposed MSMTRIU-NET model is advantageous for better distinguishing rice cultivation areas from background targets such as bare soil or water bodies.

## 3. Methodology

### 3.1. Architectures of MSMTRIU-NET

**Figure 5:**
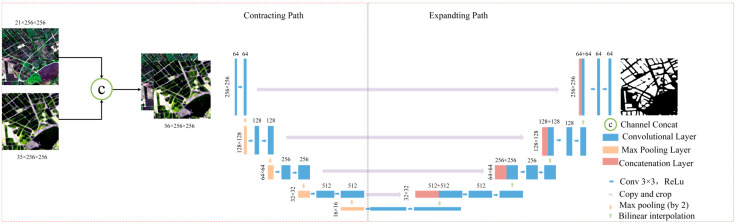
Architectures of the MSMTRIU-NET model.

The rice identification model presented in this paper is built on the U-Net network architecture [28,29]. To accurately differentiate rice from other objects with similar spectral and scattering characteristics during specific time periods each year, the model utilizes multi-temporal and multi-source input data, connecting these various data channels through channel concatenation. The model architecture, illustrated in Figure 5, consists of two primary components: the Contracting Path and the Expanding Path. In the Contracting Path, the input dataset undergoes two consecutive 3 × 3 convolution operations and a ReLU activation function, followed by 2 × 2 max pooling for down-sampling. This step progressively reduces the spatial dimensions while increasing the number of feature channels, allowing the extraction of multi-scale features. In the Expanding Path, spatial dimensions are progressively restored via up-sampling and additional convolution operations. Feature maps from the Contracting Path are fused with those in the Expanding Path using the copy and crop method, ensuring the preservation of crucial details. This encoder-decoder structure enhances the model’s ability to achieve precise image segmentation, improving overall segmentation accuracy.

### 3.2. Model Training Process

**Figure 6:**
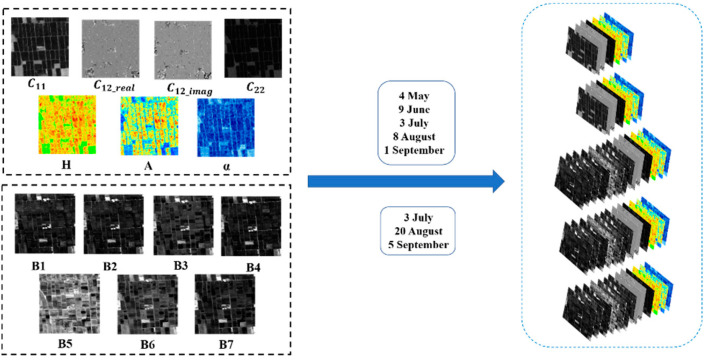
The Composition of Multi-source and Multi-temporal Datasets.

After image preprocessing, the spectral information (B1~B7) of Landsat-8 images and elements of the polarization covariance matrix in Sentinel-1 data, along with the H/A/Alpha polarimetric decomposition parameters, were all integrated within the model for training. In other words, the model input comprises 56 data channels derived from eight images, as shown in Figure 6. To train the network for the identification of rice cultivation areas, this study selected 400 samples with a size of 256 × 256 pixels. The non-rice cultivation area and rice cultivation area had the ratio of 3:1. To mitigate the overfitting problem during training, data augmentation was employed. In image classification tasks, data augmentation involved operations such as rotation, translation, and scaling applied to the original images, generating a series of new images. The augmentation approach can enhance the model’s generalization ability and robustness, even with limited data. The MSMTRIU-NET model utilized in this study applied random vertical and horizontal flips to all data during training, increasing the number of samples to 1200. The samples were randomly partitioned into three distinct sets—specifically, a training set, a validation set, and a testing set, adhering to a proportion of 5:2:3, to ensure a balanced and representative distribution for model training and evaluation.

We provided the well-trained MSMTRIU-NET model for reproducing our work at https://github.com/lile13955267907/MSMT-RIUN, accessed on 19 September 2024.

### 3.3. Experimental Setup and Quantitative Evaluation Metrics

The specific configurations used in all experiments in this study are as follows:

- Processor: 11th Gen Intel^®^ Core™ i7-11700 @ 2.50 GHz (Creator: AMD (Advanced Micro Devices, Inc.); Location: Santa Clara, CA, USA)—we utilized a high-performance hardware configuration to ensure dependable computing power.
- Memory: 32 GB—we upgraded the system to RAM in 32 GB, ensuring ample resources for processing, to eliminate any memory limitations while training large-scale deep learning models.
- Graphics Card: NVIDIA GeForce RTX 3090 (Creator: NVIDIA Corporation; Location: Santa Clara, CA, USA)—the graphics card provided out-standing computational performance and parallel processing capabilities, leading to a significant enhancement in training efficiency.

We used Python 3.8 as the programming language for writing code and developing software. PyCharm 2022.01.01 served as an integrated development environment for writing, debugging, and running Python code. For model construction and training, we utilized PyTorch 1.12.1, a widely used neural network library renowned for its comprehensive capabilities and user-friendly interface, significantly simplifying the process of efficient model design and training.

The samples in this study were generated through manual selection from the dataset, and GIS experts conducted sampling in the relevant subregions to ensure the accuracy and representativeness of the data. For the analysis of the results, this paper introduces four widely used quantitative evaluation metrics: Overall Accuracy, precision, recall, and F1-Score, aiming to provide an objective and comprehensive evaluation of the various methods. Among them, OA and F1 are more comprehensive indicators, providing a more objective and holistic assessment of the classifier’s performance [30,31,32,33].

The overall accuracy indicates the ratio of correctly predicted samples by the model to the total number of samples, and the formula is given below:

(7) $$OA=\frac{TP+TN}{TP+FP+FN+TN}$$

where *TP*, *TN*, *FP*, and *FN* represent the true class, true negative class, false positive class, and false negative class, respectively.

The precision represents the fraction of correct predictions over the total number of predictions that are positive, and the formula is as follows:

(8) $$Precision=\frac{TP}{TP+FP}$$

Recall, defined as the proportion of correctly predicted positives among all actual positives, is formulated as

(9) $$Recall=\frac{TP}{TP+FN}$$

The F1-score, as a comprehensive metric, balances the influence of precision and recall, providing a more comprehensive evaluation of the classifier, given by

(10) $$F1=\frac{2*Precision*Recall}{Precision+Recall}$$

## 4. Experimental Results

### 4.1. Ablation Experiments Using Different Datasets

**Figure 7:**
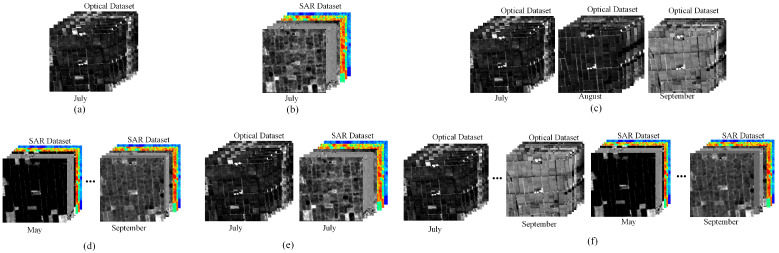
Six types of datasets for comparison: (**a**) Single-temporal optical dataset on 3 July; (**b**) Single-temporal SAR dataset on 3 July; (**c**) Multi-temporal optical dataset; (**d**) Multi-temporal SAR dataset; (**e**) Single-temporal optical and SAR dataset; (**f**) Multi-source and multi-temporal dataset.

In the proposed MSMTRIU-NET method, multi-temporal Landsat 8 and Sentinel-1 data from the study area are input into the network. The dataset comprised parameters such as spectral, intensity, phase, and the polarimetric decomposition from various rice growth stages. To showcase the advantages of incorporating multi-temporal and multi-source data, this study created the other five types of datasets (Figure 7): a single-temporal optical dataset, a single-temporal SAR dataset, a multi-temporal optical dataset, a multi-temporal SAR dataset, and a single-temporal optical and SAR dataset.

**Figure 8:**
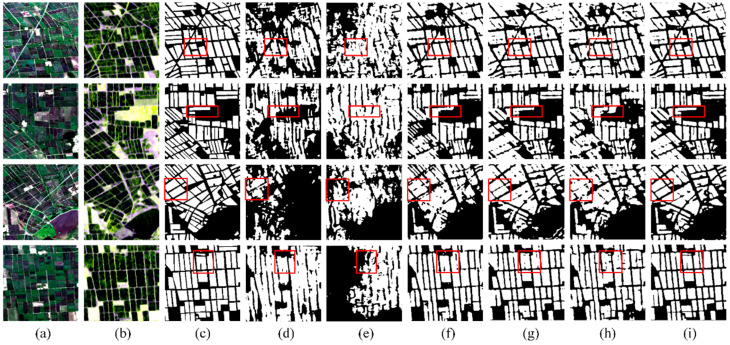
The MSMTRIU-NET’s classification maps for various datasets: (**a**) Landsat-8 optical images; (**b**) Sentinel-1 SAR images; (**c**) Label images; (**d**) Single-temporal optical images; (**e**) Single-temporal SAR images; (**f**) Multi-temporal optical images; (**g**) Multi-temporal SAR imagery; (**h**) Single-temporal optical and SAR images; (**i**) Multi-source and multi-temporal images.

Figure 8 shows the experimental results and Table 4 shows the quantitative evaluation results for the model trained by each dataset. First of all, the classification result using multi-source and multi-temporal datasets achieves the highest accuracies, while the classification accuracies using single-temporal and single-source datasets are the lowest. Given a certain image source, we can observe that using images from multiple growth stages of rice can improve the classification performance. For both single-temporal and multi-temporal datasets, we can see that the classification accuracies using a multi-source image are much higher, indicating the superiority of using both optical and SAR images in identifying rice planting areas. We notice that, for single-temporal datasets, higher classification accuracies are obtained using an optical image than using an SAR image. However, for multi-temporal datasets, higher classification accuracies are obtained using SAR images. The main reason is that only three optical images from two growth stages of rice are used in the multi-temporal optical dataset, while five SAR images from four growth stages of rice are used in the multi-temporal SAR dataset. Comparing the classification maps generated by the models using different datasets, we observe that utilizing multi-source and multi-temporal datasets leads to superior image segmentation effects, which were closest to the results of the visual interpretation results. The classification maps using the single-temporal and single-source datasets are much more fragmented due to the serious misclassification problem. In contrast, by using multi-temporal or multi-source datasets, the field blocks in the maps become more continuous.

**Figure 9:**
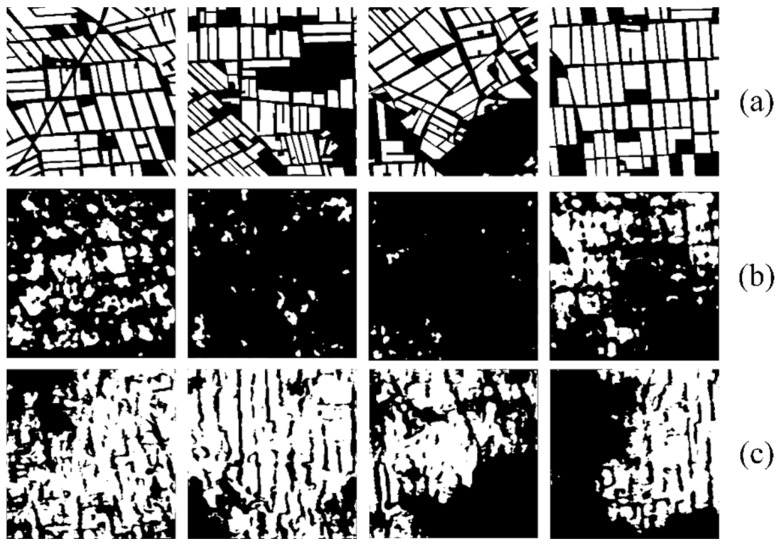
A sensitive analysis on the H/A/Alpha features in SAR data: (**a**) Label image; (**b**) Classification results excluding the H/A/Alpha features; (**c**) Classification results including the H/A/Alpha features.

A sensitive analysis of the H/A/Alpha features in SAR data was made. In Figure 9, the full feature dataset using seven features from the July SAR data is compared with the partial feature dataset that excludes the H/A/Alpha features. It can be observed in Table 5 that fully utilizing more polarimetric information in SAR data can effectively improve the accuracy of rice identification.

### 4.2. Comparison Between Different Methods

To showcase the superior capability of MSMTRIU-NET proposed in this study, a comparative analysis was undertaken to compare the model with two traditional supervised classification methods, namely, Random Forest (RF) [34] and Support Vector Machine [35], alongside Fully Convolutional Network (FCN) [36] and DeepLab v3+ [37].

**Figure 10:**
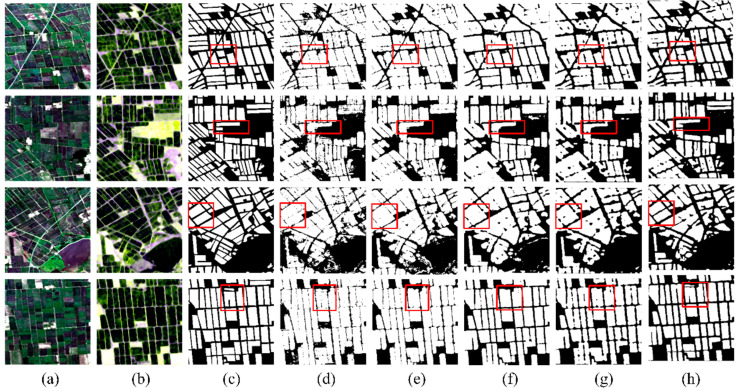
Classification maps of the various methods: (**a**) Landsat-8 optical images; (**b**) Sentinel-1 SAR images; (**c**) Label images; (**d**) SVM; (**e**) RF; (**f**) FCN; (**g**) DeepLabv3+; (**h**) MSMTRIU-NET.

In Figure 10, we present the classification results of different algorithms using multi-temporal and multi-source datasets, while Table 6 provides the quantitative evaluation results. It is evident that the recognition accuracy of traditional supervised classification methods is inferior to that of semantic segmentation algorithms. Meanwhile, the MSMTRIU-NET model demonstrated superior overall performance, surpassing other models significantly in F1-scores. The SVM classifier has the lowest accuracy, achieving an F1-score below 80%. Although RF and FCN have high recall rates, this means that while these methods can identify most of the rice, they often come with a higher false-positive rate. This indicates that they tend to mistakenly classify many non-rice pixels as rice in the rice recognition task, resulting in low OA and Precision values. The MSMTRIU-NET model effectively reduced the influence of image noise and comprehensively considered contextual semantic information, leading to balance across all metrics. The overall F1-score exhibited excellent performance, significantly enhancing the accuracy of identifying rice cultivation areas.

It can be observed that the recognition outcomes obtained through the SVM and RF classifiers revealed numerous regions with indistinct boundaries, thereby significantly affecting the overall accuracy of classification. A number of isolated pixels are misclassified as rice by SVM and RF classifiers, which are mainly affected by the coherent speckle noise in PolSAR images. This highlights that traditional interpretation methods only consider low-level features of the targets, neglecting contextual semantic information and high-level structural information in the image. For semantic segmentation algorithms, they can provide more accurate classification results by integrating multiple feature information and reducing noise, making themselves suitable for scenarios requiring high-precision identification. FCN, DeepLabv3+, and MSMTRIU-NET can capture contextual information of images and use the rich feature information from Landsat-8 and PolSAR data, thereby enabling the clearer segmentation of rice cultivation area boundaries. Overall, the classification outcomes from the MSMTRIU-NET model exhibited clearer boundaries between rice cultivation areas and surrounding regions, with internal segments more closely aligning with visual interpretations, thereby providing a more accurate representation of the real scenario in rice cultivation.

## 5. Discussion

**Figure 11:**
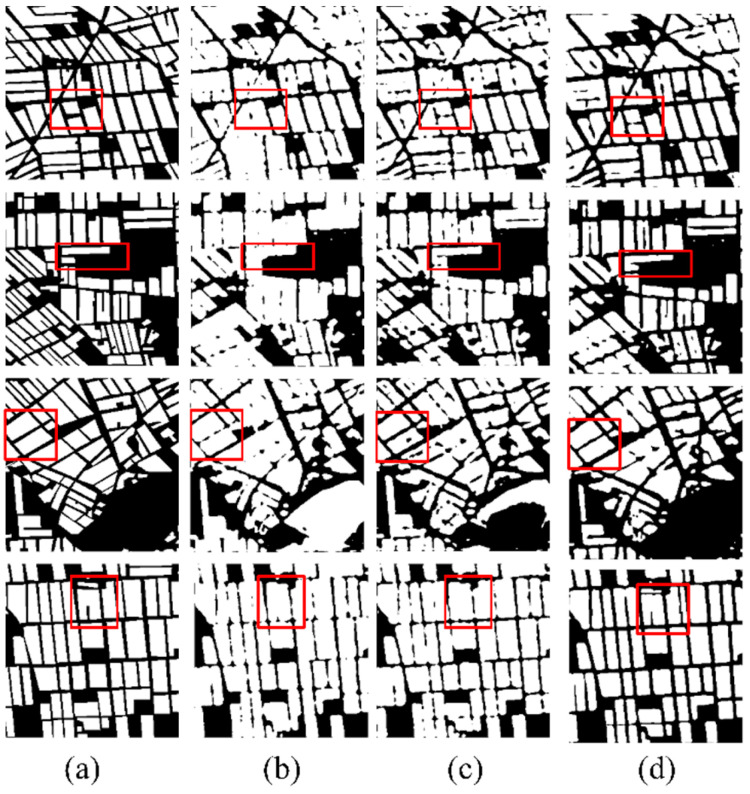
Classification results using different convolutional kernel sizes: (**a**) Label image; (**b**) 7 × 7 convolutional kernel; (**c**) 5 × 5 convolutional kernel; (**d**) 3 × 3 convolutional kernel.

The convolutional kernel size is a key parameter in the deep learning-based models. We conducted some experiments to evaluate the performances of the proposed model using different convolutional kernel sizes. The results are presented in Figure 11 and Table 7. As shown, the 3 × 3 convolutional kernel yields more desirable results for the image segmentation task. In Figure 11, the segmentation continuity achieved by the 3 × 3 kernel surpasses that of larger kernels, and the 3 × 3 kernel enables smoother and more continuous boundaries. The assessment values in Table 7 further illustrate that, as the kernel size decreases from 7 × 7 to 3 × 3, the model’s performance progressively improves. These findings support the conclusion that the 3 × 3 kernel is optimal for the proposed model.

**Figure 12:**
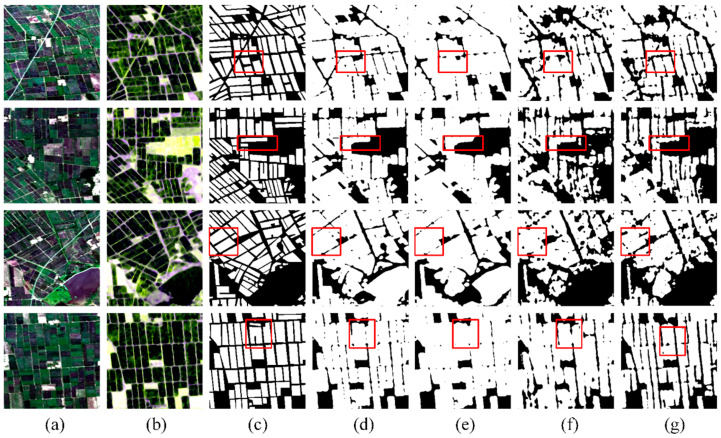
Classification maps using datasets on different stages: (**a**) Landsat-8 optical images; (**b**) Sentinel-1 SAR images; (**c**) Label images; (**d**) Classification results on the seeding stage; (**e**) Classification results on the tillering stage; (**f**) Classification results on the heading stage; (**g**) Classification results on the maturity stage.

We have also investigated the importance of datasets regarding different stages for rice identification. The results are presented in Figure 12 and Table 8. The findings indicate that the identification accuracies are highest during the maturity stage, likely due to the unique spectral reflectance and radar scattering characteristics of mature rice. Specifically, the high near-infrared reflectance and enhanced volume scattering in the PolSAR images play a key role in distinguishing rice from other crops. Although fewer images were available for the seeding stage, the model still achieved relatively good identification performance. This may be attributed to the distinct waterlogged signals during the early growth stages of rice, which differentiate it from other crops in terms of image features. Therefore, future research could benefit significantly from collecting more observational data during key growth stages, which would greatly enhance the accuracy of rice identification.

## 6. Conclusions

In remote sensing images, rice cultivation areas exhibit typical continuity and environmental complexity, making it challenging to accurately grasp the distribution of rice. Using multi-source and multi-temporal imageries enabled the accurate monitoring of these areas. This study focused on the Sanjiang Plain and proposed a deep learning model for rice cultivation area detection based on multi-source and multi-temporal imagery. The model integrated rich reflectance information from optical data and rich polarization information (including intensity, phase, and polarization decomposition parameters) from PolSAR data, achieving optimal rice identification accuracy. In the experimental part, the classification performance using single-temporal or single-source datasets is compared with that using multi-source and multi-temporal datasets, and comparisons between the proposed method and traditional supervised classification methods, as well as the classic semantic segmentation algorithm, are made. Quantitative and qualitative results demonstrate the significant advantages of the proposed model. The work in this study is applicable for tracking changes in rice cultivation areas and provides technical and theoretical support for paddy rice identification as well as decisions related to agricultural sustainability.

Despite successfully integrating two sensor modalities and utilizing multiple key phenological images of rice, this study still has some limitations. First, the limitations primarily stem from the absence of optical data during certain periods. Due to weather conditions, optical imagery in certain stages could not be obtained, resulting in an insufficient representation of rice growth stages during the seedling and tillering stages, which in turn affected the model’s recognition performance. Future research will aim to address these gaps by integrating other remote sensing data sources to compensate for the information from the missing optical data. Additionally, this study focused on a specific region and only analyzed data from 2020, without a thorough exploration of other years or more complex rice cultivation areas. Therefore, the applicability and robustness of the model in different cultivation systems and geographical environments still require further validation. Future work will also study the issue of refining the model’s architecture to improve the identifying precision.

## Figures and Tables

**Table 1:** The imaging parameters of the Landsat-8 image.

Sensor	Band Names	Spectral Band	Wavelength Range/um	SNR	Spatial Resolution/m
OLI	Coastal aerosol	B1	0.43~0.45	130	30
	Blue	B2	0.45~0.51	130	
	Green	B3	0.53~0.59	100	
	Red	B4	0.64~0.67	90	
	NIR	B5	0.85~0.88	90	
	SWIR1	B6	1.57~1.65	100	
	SWIR2	B7	2.11~2.29	100	

**Table 2:** The basic product information of Sentinel-1.

Sensor Parameter	Description
Polarimetric mode	Dual (VV + VH)
Incidence angle	20–46°
Swath	250 km
Spatial resolution	5 × 20 m
Band	C
Mode	IW
Product type	SLC
Product level	Level-1

**Table 3:** Acquisition time of Landsat-8 and Sentinel-1 images.

Data Source	Acquisition Time	Season	Rice Phenological Periods
Landsat-8	3 July	Summer	Heading stage
	20 August		
	5 September	Autumn	Maturity stage
Sentinel-1 A/B	4 May	Spring	Seeding stage
	9 June	Summer	Tillering stage
	3 July		Heading stage
	8 August		
	1 September	Autumn	Maturity stage

**Table 4:** Four evaluation metrics resulting from MSMTRIU-NET maps trained across various datasets.

Dataset	OA (%)	Precision (%)	Recall (%)	F1-Score (%)
The single-temporal optical dataset	80.80	70.53	54.09	61.22
The single-temporal SAR dataset	78.08	62.20	55.53	58.68
The multi-temporal optical dataset	87.72	80.09	74.74	77.33
The multi-temporal SAR dataset	93.11	85.68	90.57	88.06
The single-temporal optical and SAR dataset	87.95	78.94	77.73	78.33
The multi-source and multi-temporal dataset	95.71	91.47	89.90	90.68

**Table 5:** Four evaluation metrics resulting from different feature dataset.

Dataset	OA (%)	Precision (%)	Recall (%)	F1-Score (%)
Without H/A/Alpha features	74.30	73.93	12.84	21.88
With H/A/Alpha features	78.08	62.20	55.53	58.68

**Table 6:** Four evaluation metrics resulting from MSMTRIU-NET maps trained across various methods.

Method	OA (%)	Precision (%)	Recall (%)	F1-Score (%)
SVM	86.34	68.44	90.64	77.99
RF	90.07	74.51	95.43	83.68
FCN	91.38	77.71	97.12	86.34
DeepLabv3+	92.20	82.93	90.89	86.72
MSMTRIU-NET	95.71	91.47	90.87	90.68

**Table 7:** Four evaluation metrics resulting from different convolutional kernel sizes.

Convolutional Kernel Size	OA (%)	Precision (%)	Recall (%)	F1-Score (%)
7 × 7	87.46	70.55	94.81	80.90
5 × 5	91.33	80.25	91.62	85.56
3 × 3	95.71	91.47	90.87	90.68

**Table 8:** Assessment values using different datasets.

Dataset	OA (%)	Precision (%)	Recall (%)	F1-Score (%)
Dataset on seeding stage	82.88	62.34	98.25	76.28
Dataset on tillering stage	81.63	60.53	99.01	75.13
Dataset on heading stage	86.84	73.81	82.20	77.78
Dataset on maturity stage	87.72	74.33	85.82	79.66

## Data Availability

The authors confirm that the data supporting the findings of this study are cited within the article.
